# The Amidase Domain of Lipoamidase Specifically Inactivates Lipoylated Proteins *In Vivo*

**DOI:** 10.1371/journal.pone.0007392

**Published:** 2009-10-08

**Authors:** Maroya D. Spalding, Sean T. Prigge

**Affiliations:** 1 Department of Biochemistry and Molecular Biology, Johns Hopkins Bloomberg School of Public Health, Baltimore, Maryland, United States of America; 2 Department of Molecular Microbiology and Immunology and Malaria Research Institute, Johns Hopkins Bloomberg School of Public Health, Baltimore, Maryland, United States of America; University of Wisconsin-Milwaukee, United States of America

## Abstract

**Background:**

In the 1950s, Reed and coworkers discovered an enzyme activity in *Streptococcus faecalis* (*Enterococcus faecalis*) extracts that inactivated the *Escherichia. coli* and *E. faecalis* pyruvate dehydrogenase complexes through cleavage of the lipoamide bond. The enzyme that caused this lipoamidase activity remained unidentified until Jiang and Cronan discovered the gene encoding lipoamidase (Lpa) through the screening of an expression library. Subsequent cloning and characterization of the recombinant enzyme revealed that lipoamidase is an 80 kDa protein composed of an amidase domain containing a classic Ser-Ser-Lys catalytic triad and a carboxy-terminal domain of unknown function. Here, we show that the amidase domain can be used as an *in vivo* probe which specifically inactivates lipoylated enzymes.

**Methodology/Principal Findings:**

We evaluated whether Lpa could function as an inducible probe of α-ketoacid dehydrogenase inactivation using *E. coli* as a model system. Lpa expression resulted in cleavage of lipoic acid from the three lipoylated proteins expressed in *E. coli*, but did not result in cleavage of biotin from the sole biotinylated protein, the biotin carboxyl carrier protein. When expressed in lipoylation deficient *E. coli*, Lpa is not toxic, indicating that Lpa does not interfere with any other critical metabolic pathways. When truncated to the amidase domain, Lpa retained lipoamidase activity without acquiring biotinidase activity, indicating that the carboxy-terminal domain is not essential for substrate recognition or function. Substitution of any of the three catalytic triad amino acids with alanine produced inactive Lpa proteins.

**Conclusions/Significance:**

The enzyme lipoamidase is active against a broad range of lipoylated proteins *in vivo*, but does not affect the growth of lipoylation deficient *E. coli*. Lpa can be truncated to 60% of its original size with only a partial loss of activity, resulting in a smaller probe that can be used to study the effects of α-ketoacid dehydrogenase inactivation *in vivo*.

## Introduction

The cofactor lipoic acid is necessary for the oxidative decarboxylation of 2-ketoacids, an activity essential to the tricarboxylic acid cycle, amino acid metabolism, heme biosynthesis, and other metabolic processes. Oxidative decarboxylation is carried out by large, multisubunit complexes, including pyruvate dehydrogenase (PDH), α-ketoglutarate dehydrogenase (KDH), and branched chain α-ketoacid dehydrogenase (BCDH) [1], [2]. These complexes are composed of three subunits, the E1 α-ketoacid dehydrogenase, the E2 transacetylase, and the E3 dihydrolipoyldehydrogenase [1], [3]. The carbonyl of lipoic acid is attached to the ε-amino group of specific lysine residues on the E2 subunits, forming an amide bond [4]. Lipoic acid plays two critical roles in α-ketoacid dehydrogenase complexes. It is reductively acylated by the E1 subunit and subsequently functions as a swinging arm to transfer the covalently bound acyl group to the active site of the E3 subunit [1]. A fourth enzyme complex, called the glycine cleavage complex (GCV), catalyzes similar reactions, but the nomenclature is different and the subunit containing lipoic acid is referred to as the H-protein [5], [6].

In the 1950s, while studying the role of lipoic acid in the activation of the *Streptococcus faecalis* (now *Enterococcus faecalis*) PDH, Reed and coworkers discovered a partially purified enzyme activity from *E. faecalis* that inactivated the *Escherichia coli* and *E. faecalis* PDH and caused the release of free lipoic acid [7]. Further purification and analysis of the unidentified protein, called lipoamidase (Lpa), established that the enzyme cleaved lipoic acid from α-ketoacid dehydrogenases and lipoic acid amide and ester small molecules but had little to no activity on ε-N-biotinyl-L-lysine (biotinyl-lysine), ε-N-acetyl-L-lysine, or ε-N-benzoyl-L-lysine [8]. The gene and protein that were the source of this activity remained unknown for 50 years, until Jiang and Cronan identified the Lpa gene by screening an expression library for Lpa activity [9]. The Lpa gene encodes an 80 kDa protein with an N-terminal amidase domain featuring a characteristic Ser-Ser-Lys catalytic triad and a C-terminal domain of unknown function [9]. Overexpression of Lpa in *E. coli* and purification of the enzyme to near homogeneity enabled further study of Lpa activity. The purified enzyme inactivated *E. coli* lipoyl proteins and cleaved lipoate from lipoyl-lysine. Purified Lpa also cleaved biotin from biotinyl-lysine, albeit at reduced levels compared to cleavage of lipoate from lipoyl-lysine.

Since its discovery in partially purified *E. faecalis* extracts 50 years ago, lipoamidase activity has played an important role in establishing lipoate as an essential cofactor for α-ketoacid dehydrogenase activity and in studies of *E. coli* lipoic acid biosynthesis [2]. The recent identification of the gene encoding Lpa now makes it possible to use Lpa as a probe to study the effects of α-ketoacid dehydrogenase inactivation *in vivo*. We evaluated the activity and specificity of Lpa in *E. coli* using an inducible expression system. Lpa cleaved radiolabeled lipoic acid from all three lipoylated proteins in *E. coli*, and inhibited bacterial growth. Substitution of any of the three catalytic triad amino acids to alanine yielded inactive enzyme *in vivo*. Expression of Lpa *in vivo* does not affect biotinylation of the *E. coli* acetyl-CoA carboxylase (ACCase), demonstrating that Lpa is a specific probe for lipoate disruption despite its broad substrate specificity among lipoylated proteins. Consequently, expression of Lpa in lipoate deficient cells is not toxic. Lpa retains activity and specificity when truncated to its 47 kDa amidase domain, resulting in a small, soluble probe that is amenable to heterologous expression.

## Materials and Methods

### Cloning of Lpa and Lpa mutants

All primers used for this work are listed in **Table 1**. Primers were obtained from Invitrogen, endonucleases were purchased from New England Biolabs, and all PCR reactions were performed with TurboPfu DNA polymerase (Stratagene). The gene encoding lipoamidase from *E. faecalis* strain V583 was amplified by PCR from plasmid pYFJ62 (a kind gift from John Cronan) [9] using the Lpa primers. The PCR product was digested with BamHI and SalI followed by ligation into the pLZ expression vector [10] (*MalE* gene of pMAL_cHT [11] replaced with the amino acids MRGS) to generate plasmid pMS007. Constructs expressed in the pLZ vector are produced with an N-terminal hexa-histidine tag composed of the amino acids MRGSHHHHHHEFGS. Active site and truncation mutants of Lpa were generated by site-directed mutagenesis with the QuickChange mutagenesis kit (Stratagene) using the manufacturer's directions. For the active site mutants, plasmid pMS007 was used with the primers K159A, S235A, and S259A to produce pMS012, pMS029, and pMS008, respectively. For the truncation mutants, the primers t471 and t521 were used to generate constructs that replaced amino acids 471 and 521 with stop codons, resulting in plasmids pMS009 and pMS010, respectively. The PCR products from all mutagenesis reactions were digested with DpnI and transformed into TOP10 cells (Invitrogen). Plasmids isolated from individual colonies were sequenced to confirm all lipoamidase mutations.

**Table 1:** Primer sequences.

Primer	Sequence (5′-3′)
Lpa F	GGTGGTGGATCCATGTTGGCACAAGAAAGTATACTAG
Lpa R	GGTGGTGTCGACTTATCATTTTCTAGTTTTCCTTATATAAATC
K159A 1	GGTGTGCCGCTCTTACTAGCAGGGTTAGGACAATCCTTG
K159A 2	CAAGGATTGTCCTAACCCTGCTAGTAAGAGCGGCACACC
S235A 1	TGGAATCCTAACCATTATTCAGGTGGTGCTTCAGGCGGAGCGCCGG
S235A 2	CCGGCGCTCCGCCTGAAGCACCACCTGAATAATGGTTAGGATTCCA
S259A 1	GAAGTGATGCTGGTGGCGCTATCCGCATCCCTGC
S259A 2	GCAGGGATGCGGATAGCGCCACCAGCATCACTTC
t471 1	TTACTAAAACCAGAACATGCAGCATGATCTAGAAAAATTGATCAATTGTCACCAGCAG
t471 2	CTGCTGGTGACAATTGATCAATTTTTCTAGATCATGCTGCATGTTCTGGTTTTAGTAA
t521 1	AAAGTGTACCAACTTACGTTTCAAAATAGTCTAGACCTTTGGGGATTCAATTTAATAGTG
t521 2	CACTATTAAATTGAATCCCCAAAGGTCTAGACTATTTTGAAACGTAAGTTGGTACACTTT

### Western blotting

Proteins were separated on 4–12% sodium-dodecyl sulfate polyacrylamide gels (SDS-PAGE) (Invitrogen) and transferred to nitrocellulose membranes. Membranes were probed with an antibody that specifically recognizes MRGSHis_6_ and is conjugated to horseradish peroxididase (HRP) (1∶1000, Qiagen), rabbit antiserum specific for lipoylated proteins (1∶10,000, Calbiochem), streptavidin-HRP (1∶1000, Calbiochem), or rabbit antiserum specific for *E. coli* HSP70 (1∶20,000, a gift from Roger McMacken). When necessary, HRP conjugated donkey anti-rabbit serum (1∶5000, GE healthcare) was used as a secondary probe. HRP-conjugated probes were detected with the Supersignal West Pico chemiluminescence kit (Pierce). Blots that were subject to more than one western blot experiment were stripped with 4% trichloroacetic acid twice for 15 minutes between analyses. Densitometry analyses were conducted in ImageJ, and lipoylation and biotinylation levels were determined by normalizing signals for these cofactors to anti-HSP70 signal. Relative lipoylation levels were obtained by dividing the normalized signal for Lpa samples by the normalized signal for samples expressing vector alone. Error bars represent the standard error of the mean between three or more independent western blots.

### Uptake and incorporation of ^35^S-lipoic acid in cells expressing Lpa and Lpa S259A

The plasmids pMS007 and pMS008 were transformed into KER176 *E. coli* (*lipA*::TN1000dkan) [12]. The KER176 strain (a gift from John Cronan) does not contain a functional lipoate synthase, and is thus a lipoate auxotroph. *E. coli* KER176 harboring the plasmid pMS007 or pMS008 were cultured overnight in LB media supplemented with 25 ng/ml lipoic acid, 100 µg/ml carbenicillin, and 50 µg/ml kanamycin. Cells were diluted 1∶10,000 in a modified E minimal media [13] supplemented with 0.4% glucose, 1 mg/ml vitamin-free casein hydrolysate, 2 µg/ml thiamine, 7.5 µg/ml ferrous sulfate, 50 µg/ml kanamycin, and 100 µg/ml carbenicillin. Diluted cells were added 1∶50 to 0.5 ml of supplemented minimal media containing ^35^S-lipoic acid (10 ng with a specific activity of 34.2 Ci/mmol) [10] and cultured at 37°C. At OD_600_ = 0.6 protein expression was induced with 0.4 mM IPTG and the cells were cultured for an additional 10 hours at 20°C. After induction, the cultures were normalized based on optical density at 600 nm (OD_600_) and harvested by centrifugation. Cell pellets were lysed in 1x SDS-PAGE sample buffer by three cycles of heating for 5 minutes at 95°C followed by 30 seconds of vortexing. Lipoic acid uptake and retention was measured by scintillation counting of cell lysates. Lipoic acid incorporation was assessed by separating the cell lysates on a 4–12% gradient SDS-PAGE gel followed by transfer to nitrocellulose membrane and analysis by autoradiography. Differences in levels of lipoic acid incorporation into the PDH, KDH, and H-protein in cells expressing Lpa compared to cells expressing Lpa S259A were determined by densitometry analysis performed with ImageJ.

### Assay of Lpa expression and its effect on growth, lipoylation, and biotinylation

Several growth, lipoylation, and biotinylation experiments were conducted using BL21-Star(DE3) *E. coli* (Invitrogen) containing the pRIL plasmid isolated from BL21-CodonPlus(DE3) cells (Stratagene). These cells were transformed with the empty expression vector pLZ, or with pLZ encoding wild type lipoamidase (pMS007), active site point mutants (pMS008, pMS012, and pMS029), or the truncation mutants (pMS009 and pMS010). As additional controls, cells were also prepared with two plasmids (pSP010 and pMS002) encoding unrelated genes (malonyl-CoA:ACP acyltransferase (MCAT) [14] and ketoacyl-ACP synthase II (KASII) [15] from *Plasmodium falciparum*) in the pMALcHT expression vector [11]. This vector expresses MBP (Maltose Binding Protein) fusion proteins, but otherwise has an identical plasmid backbone to that of pLZ. Transformed BL21-Star(DE3) cells were grown at 37°C and selected on LB-agar plates containing 100 µg/ml carbenicillin and 35 µg/ml chloramphenicol. Colonies were selected in triplicate for each construct and cultures were grown overnight at 37°C with shaking in LB media containing 100 µg/ml carbenicillin and 35 µg/ml chloramphenicol. The cultures were then diluted to OD_600_ = 0.1 in fresh LB medium with antibiotics and grown for one hour at 37°C. Protein expression was induced by the addition of 0.4 mM IPTG followed by 10 hours growth at 37°C or 20°C. The OD_600_ of the cultures was measured at 0, 2, 4, 6, and 10 hours post-induction. For each triplicate set of cultures, the culture with median growth at 10 hours was selected for analysis of protein lipoylation and biotinylation by western blot (described above). The volume of cells harvested for analysis was normalized to 400 µl of the culture with the lowest OD_600_. The cell pellets were resuspended in 100 µl of 1X SDS-PAGE sample buffer and lysed as previously described. The cell lysate was then diluted 1∶10 in sample buffer and the equivalent of 1 µl of culture was run per lane.

### Comparison of ^35^S-lipoic acid incorporation in cells expressing Lpa active site mutants

Plasmids encoding mutant lipoamidase (pMS008, pMS012, and pMS029) were transformed into KER176 *E. coli* to determine whether any of these mutants display detectable lipoamidase activity. As controls, empty expression vector pLZ and pSP010 encoding *P. falciparum* MCAT (described above) were also transformed into KER176. Transformed cells were grown overnight in LB medium supplemented with 10 ng/ml lipoic acid, 100 µg/ml carbenicillin, and 50 µg/ml kanamycin. Cells were diluted 1∶500 in a modified E minimal media [13] supplemented with 0.4% glucose, 1 mg/ml vitamin-free casein hydrolysate, 2 µg/ml thiamine, 7.5 µg/ml ferrous sulfate, 50 µg/ml kanamycin, and 100 µg/ml carbenicillin. Diluted cells were added 1∶50 to 0.5 ml of supplemented minimal media containing ^35^S-lipoic acid (7 ng with a specific activity of 34 Ci/mmol) [10] and cultured at 37°C. At OD_600_ = 0.4–0.6 protein expression was induced with 0.4 mM IPTG and the cells were cultured for an additional 6 hours at 20°C. After induction, the cultures were normalized based on optical density at 600 nm (OD_600_) and harvested by centrifugation. Cell pellets were lysed in 1x SDS-PAGE sample buffer by three cycles of heating for 5 minutes at 95°C followed by 30 seconds of vortexing. Lipoic acid incorporation was assessed by separating the cell lysates on a 4–12% gradient SDS-PAGE gel followed by transfer to nitrocellulose membrane and analysis by autoradiography. The incorporation of ^35^S-lipoic acid into the *E. coli* PDH, KDH, and H-protein was determined by densitometry analysis performed with ImageJ.

### Lpa expression in TM136 strain *E. coli*

Plasmids encoding wild type (pMS007) and active site mutant (pMS029 and pMS008) Lpa were transformed into lipoylation deficient TM136 strain *E. coli* [16]. Colonies were selected on LB-agar plates supplemented with 2% glucose, 5 mM sodium acetate, 5 mM sodium succinate, 1 g/L vitamin-free casein hydrolysate and antibiotics (50 µg/ml kanamycin, 7.5 µg/ml tetracycline, and 100 µg/ml carbenicillin). Three colonies were chosen from each plate and grown to mid-log phase at 37°C in TM136 medium (LB supplemented with 2% glucose, 5 mM sodium acetate, 5 mM sodium succinate, 1 g/L vitamin-free casein hydrolysate, 50 µg/ml kanamycin, 7.5 µg/ml tetracycline, and 100 µg/ml carbenicillin). These cultures were diluted in the same medium to an OD_600_ of 0.05 and were each divided into four replicate cultures. These were allowed to grow at 37°C for 45 minutes after which the four replicates were used to initiate the growth experiment. Two replicates were maintained at 37°C and IPTG was added to one at a final concentration of 0.4 mM. The other two replicates were maintained at 20°C (with and without IPTG). Optical density measurements were made at this time point (t = 0) and every two hours thereafter to track the growth of these cultures.

### Assay of lipoamidase solubility

The expression and solubility of wild type Lpa and the truncation mutants Lpa_t471_ and Lpa_t521_ were assessed in BL21-Star(DE3) cells containing the plasmid pRIL (described above). Cultures harboring each construct were grown to an OD_600_ of 0.6 at 37°C in 100 ml of LB medium containing 100 µg/ml carbenicillin and 35 µg/ml chloramphenicol. At this point, protein expression was induced with 0.4 mM IPTG and each culture was split with 50 ml maintained at 37°C for four hours, and 50 ml maintained at 20°C for 10 hours. At the end of the induction period, 10 ml was removed from the culture with the lowest OD_600_ and harvested by centrifugation. Equivalent amounts (based on optical density) were harvested from the other cultures, and the cell pellets were lysed by incubation in 1 ml of Bug Buster reagent (Novagen) containing 1 mg/ml lysozme and 10 µg/ml DNaseI for 10 minutes at room temperature. The insoluble fraction was isolated by centrifugation of whole cell lysate at 16,000 *g* for 10 minutes and resolubilized in 1 ml of 6 M urea. Protein samples containing 0.5 µl of the soluble or insoluble fraction were separated by SDS-PAGE and analyzed by anti-MRGSHis_6_ western blot, as described above.

### Analysis of lipoylation and biotinylation sites

The amino acid sequences of lipoylated and biotinylated proteins in *E. coli*, *Saccharomyces cerevisiae*, *Homo sapiens*, and *P. falciparum* were obtained from the NCBI database. The bacterial genome resource J. Craig Venter Institute was then used to identify lipoylated proteins in *E. faecalis* through BLAST homology searches with the *E. coli* and *S. cerevisiae* lipoylated proteins. Amino acid sequences were aligned using CLUSTAL W [17].

## Results

### Lpa expression *in vivo*

**Figure 1:**
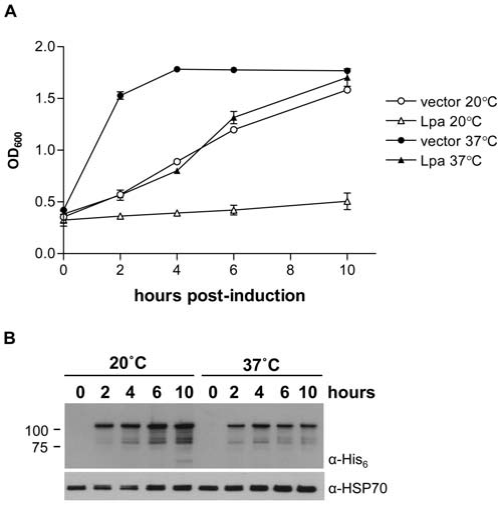
Lpa expression at 20 and 37°C. (A) Growth assay of *E. coli* cells expressing lipoamidase at 20°C (open triangles) and 37°C (closed triangles) relative to cells containing vector alone at 20°C (open circles) and 37°C (closed circles). The OD_600_ of cultures was measured at the time of induction with IPTG and then 2, 4, 6, and 10 hours post-induction. Error bars represent the standard deviation of three replicates. (B) Anti-His western blot of cells expressing Lpa at 20°C and 37°C. Samples were taken from cultures at the time of induction and at 2, 4, 6, and 10 hours post-induction.

We cloned the V583 lipoamidase gene into a derivative of the pMAL vector that produces His_6_-Lpa and expressed the enzyme in BL21-Star(DE3) cells. We observed that transformed cells were viable when grown at 37°C but not after overnight storage at 4°C, perhaps due to increased sensitivity of cells to leaky expression of the toxic *lpa* gene at low temperature. To determine if Lpa expressed *in vivo* is active at the physiologic temperature 37°C and to compare the effects of lipoamidase expression at physiological and sub-physiological temperatures, cell growth and lipoamidase levels were tracked over the course of ten-hour inductions at 37°C and 20°C. *E. coli* expressing Lpa at 37°C exhibited slow growth during the first four hours of induction but attained a cell density similar to that of the control culture by the end of the induction period (**Figure 1A**). In contrast, cultures maintained at 20°C exhibited a severe growth defect over the course of the ten hour induction period (**Figure 1A**). Western blot analysis of whole cell lysate shows that Lpa production is sustained throughout the 20°C induction and peaks at ten hours. At 37°C, production peaks at four hours, and the decline in Lpa production after this time point coincides with an increase in cell growth (**Figure 1B**). Thus, Lpa is expressed in active form at both 37°C and 20°C, and its activity at a range of temperatures suggests that it could be used as a probe of *in vivo* lipoyl protein inactivation in organisms other than *E. coli*. Because the accumulation of Lpa over a ten hour induction period at 20°C results in an extended period of slow *E. coli* growth, in subsequent assays we expressed Lpa at 20°C to take advantage of the longer window of observation at this temperature.

### Activity of Lpa *in vivo*

**Figure 2:**
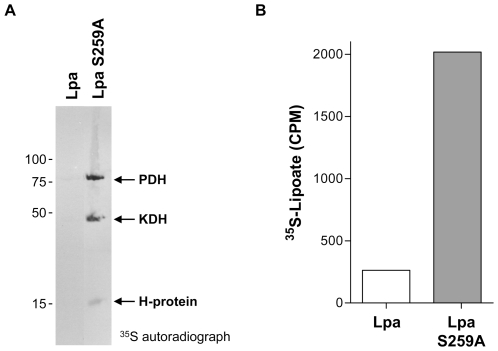
Uptake and incorporation of ^35^S-lipoic acid in the presence of Lpa. (A) KER176 cells that are auxotrophic for lipoic acid were transformed with plasmids encoding wild type Lpa (Lpa) or the active site nucleophile mutant Lpa S259A and were grown in minimal medium supplemented with ^35^S-lipoic acid. After a 10 hour induction at 20°C, cell samples were normalized by OD_600_ and protein extracts were separated by SDS-PAGE and analyzed by autoradiography. The assignment of the labeled species to the three lipoylated proteins in *E. coli*, the PDH, KDH, and H-protein, is indicated. (B) Scintillation counting was used to quantify ^35^S-lipoic acid taken up by the KER176 cultures shown in Figure 2A. Counts per minute (CPM) correspond to the uptake of cells from 5 µl of culture with an OD_600_ of 1.1.

The biological role of Lpa in *E. faecalis* may rely on its ability to recognize exogenous lipoylated peptides or proteins. We tested the activity of Lpa against the three lipoylated proteins found in *E. coli* (the H-protein of the glycine cleavage complex and the E2 subunits of pyruvate dehydrogenase and alpha-ketoglutarate dehydrogenase) to determine whether the enzyme has a broad specificity for lipoylated proteins. The lipoate auxotroph strain KER176 was transformed with plasmids encoding wild type Lpa (Lpa) or the active site nucleophile mutant Lpa S259A and grown in a minimal media containing ^35^S-lipoate as the sole source of lipoate, resulting in the incorporation of radiolabeled lipoate in the three *E. coli* proteins. At an OD_600_ of 0.6, expression of Lpa and Lpa S259A was induced with IPTG. After the induction period, cell lysates were separated by SDS-PAGE and analyzed by autoradiography. Incorporation of radiolabeled lipoate into the three lipoylated enzymes in *E. coli* was greatly reduced in cells expressing Lpa compared to cells expressing Lpa S259A (**Figure 2A**). Analysis of the autoradiograph by densitometry shows that lipoylation of the PDH E2, KDH E2, and H-protein in cells expressing Lpa is reduced to 2%, 1%, and less than 1%, respectively, of lipoylation in cells expressing Lpa S259A.

### Fate of cleaved lipoic acid

Prior evidence suggests that *E. coli* does not maintain large intracellular stores of free lipoate. Herbert and Guest measured PDH and KDH activities in *E. coli* extracts and found that these enzymatic activities correlated well with the total cellular content of lipoic acid, even when an excess of lipoic acid was added to the growth medium [18]. These results suggest that either *E. coli* limits the uptake of lipoic acid, or that the bacterium fails to retain excess lipoic acid that is not needed to activate the two ketoacid dehydrogenases. By densitometry analysis of the western blot in **Figure 2A**, we determined that the total incorporation of lipoate into proteins in cells expressing Lpa is 1% of that in cells expressing Lpa S259A. We also measured ^35^S-lipoate in the lysates of KER176 cells expressing either Lpa or Lpa S259A, and found that cells expressing the active enzyme contained 87% less ^35^S-lipoate than cells expressing the inactive enzyme (**Figure 2B**). Together, these results indicate that as Lpa cleaves lipoate from lipoylated proteins, the free acid fails to accumulate in the cell.

### Effect of Lpa on biotinylation

**Figure 3:**
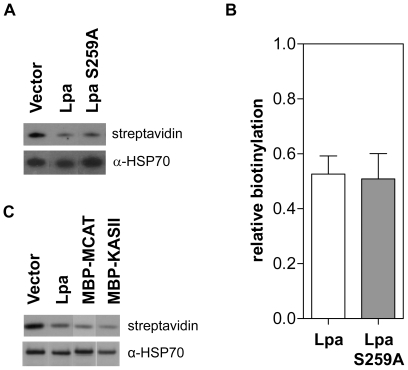
Effect of protein expression on biotinylation. (A) Affinity blot analysis of biotinylation in *E. coli* BL21 cells expressing Lpa and Lpa S259A. Samples were normalized by OD_600_ and the protein extracts from whole cell lysate were separated by SDS-PAGE and blotted onto nitrocellulose. The biotinylation status of the BCCP, the sole biotinylated protein in *E. coli*, was analyzed by streptavidin-HRP western blot. (B) Densitometry analysis of the biotinylation level in cells expressing Lpa and Lpa S259A. Streptavidin-HRP signal was normalized to the HSP70 loading control signal for each sample. Within each blot, the fraction of biotinylation relative to cells expressing vector alone was determined. Error bars represent the SEM for fraction of biotinylation for three independent western blots. (C) Affinity blot analysis of biotinylation in *E. coli* BL21 cells expressing Lpa and the unrelated proteins MBP-MCAT and MBP-KASII. Samples were analyzed by the methods described in part A of this figure.

The broad substrate specificity of Lpa raises the possibility that it could also cleave the amide bond linking biotin to the biotin-carboxyl-carrier protein (BCCP) subunit of the acetyl-CoA carboxylase (ACCase) found in *E. faecalis* and *E. coli*. In a bioassay, Lpa was observed to cleave biotin from biotinyl-lysine [9]; therefore, we sought to determine whether Lpa also cleaves biotin from proteins *in vivo*. The biotinylation status of the *E. coli* BCCP (the sole biotinylated protein in *E. coli*) was monitored with streptavidin-HRP. Cells expressing both Lpa and Lpa S259A appeared to contain similar levels of biotinylated BCCP; however, this level was markedly less than in cells expressing empty vector (**Figure 3A**). Analysis of western blots by densitometry shows that biotinylation of the BCCP in cells expressing Lpa and those expressing Lpa S259A is approximately half the level of cells expressing the empty vector, but that there is no significant difference in biotinylation levels between cells expressing active and inactive lipoamidase (**Figure 3B**). Expression of the BCCP in *E. coli* is dependent on the cellular growth rate [19]; therefore, the apparent difference in levels of biotinyl-BCCP is likely derived from lower BCCP expression in cells expressing Lpa compared to cells expressing the small peptide produced by the empty vector. In general, the metabolic burden of protein over-expression typically results in a significantly decreased replication rate. To confirm that the decreased biotinylation of the BCCP was derived from protein over-expression and was not Lpa-specific, we evaluated BCCP biotin levels after the expression of two proteins: *P. falciparum* malonyl-CoA:ACP acyltransferase (MCAT) and *P. falciparum* ketoacyl-ACP synthase II (KASII). In both cases, we observed a reduction in biotin levels similar to that observed in the cells expressing Lpa (**Figure 3C**). Thus, it appears that decreased biotinylation of BCCP is an artifact of protein over-expression and that lipoamidase does not have significant biotinidase activity against proteins *in vivo*.

### Effect of Lpa and active site mutants on growth, lipoylation, and biotinylation *in vivo*

**Figure 4:**
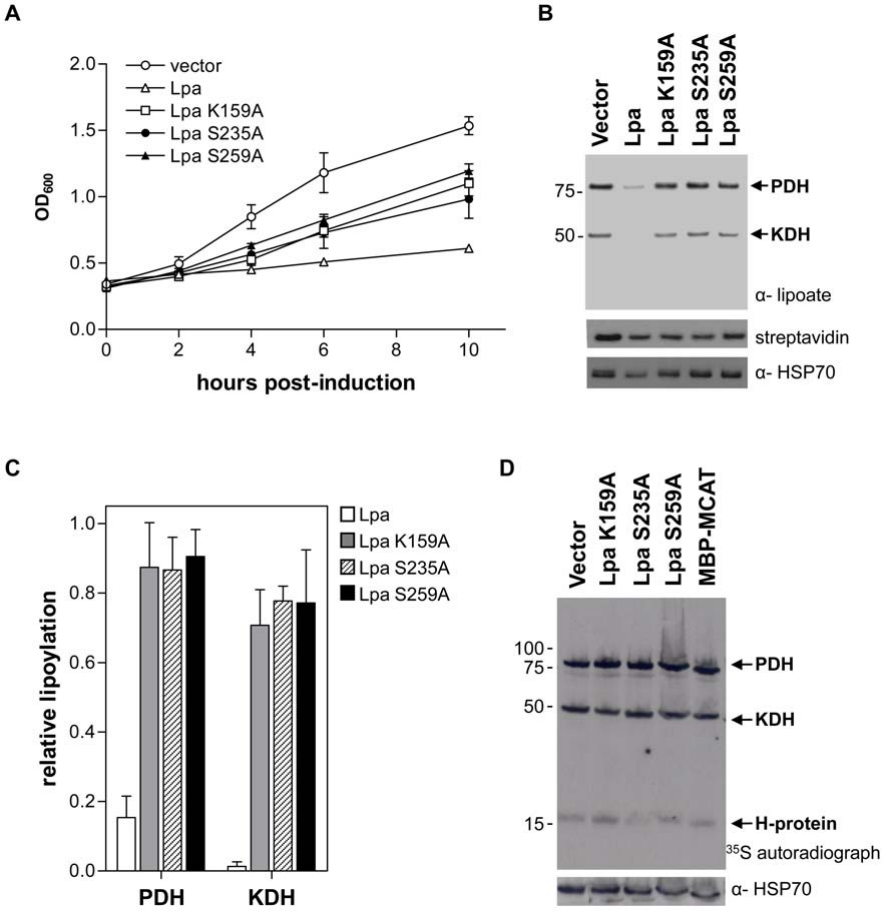
Growth, lipoylation, and biotinylation of cells expressing Lpa and Lpa active site mutants. (A) Growth assay of *E. coli* BL21 cells expressing Lpa (open triangles) compared to vector alone (open circles) and the three active site mutants: Lpa K159A (open squares), Lpa S235A (closed circles), and Lpa S259A (closed triangles). The OD_600_ of cultures was measured at the time of induction with IPTG and at 2, 4, 6, and 10 hours post-induction. Error bars represent the standard deviation of three replicates from a representative growth assay. (B) Anti-lipoic acid and streptavidin affinity blots to determine levels of PDH and KDH lipoylation and BCCP biotinylation in *E. coli* expressing empty vector, Lpa, and Lpa active site mutants. (C) Densitometry analysis of PDH and KDH lipoylation in cells expressing Lpa and Lpa active site mutants. To determine the level of KDH and PDH lipoylation, the anti-lipoic acid signal was normalized to the anti-HSP70 loading control signal. The fraction of lipoylation in cells expressing Lpa or the Lpa active site mutants relative to cells expressing vector alone was then determined. Error bars represent the SEM for the fraction of lipoylation for three independent western blots. (D) Autoradiograph of KER176 cells expressing vector alone, the Lpa active site mutants, and MBP-MCAT grown in minimal medium supplemented with ^35^S-lipoic acid. After a 6 hour induction at 20°C, cell samples were normalized by OD_600_ and protein extracts were separated by SDS-PAGE and analyzed by autoradiography. Equal sample loading was assessed by anti-HSP70 western blot.

Jiang and Cronan used a bioassay with the small molecule substrate lipoyl-lysine to determine the activity of Lpa when individual residues of the catalytic triad were mutated to alanine [9]. Mutation of either of the two active site serines was found to block lipoamidase activity, while mutation of the active site lysine produced a partially active mutant. The effect of these mutations on biotinyl-lysine cleavage was not determined. We compared the effects of Lpa and the three active site mutants, the inactive Lpa S235A and Lpa S259A and the partially active mutant Lpa K159A, on growth, lipoylation, and biotinylation in *E. coli*. The growth of cells expressing Lpa was severely compromised compared to cells expressing the empty vector or the three active site mutants (**Figure 4A**). Cells expressing the mutants did not grow to the same OD_600_ as cells expressing vector alone, an anticipated effect of protein over-expression. Interestingly, the growth of cells expressing Lpa K159A did not significantly differ from that of the two inactive serine mutants.

The dramatic growth defect observed with Lpa expression is correlated with a severe decrease in lipoylation of the PDH and KDH as observed by anti-lipoate western blot (**Figure 4B**). Consistent with our previous observations, no difference in biotinylation was observed between cells expressing Lpa and any of the active site mutants (**Figure 4B**). The *E. coli* PDH contains three lipoyl domains, in contrast to the *E. coli* KDH, which contains a single lipoyl domain and thus appears lighter than the PDH band [1]. The third lipoylated protein in *E. coli*, the H-protein, is not detected by anti-lipoate western blot. Expression of wild type Lpa reduced the lipoylation of the PDH and KDH to 15% and 1%, respectively, of the levels in cells expressing empty vector (**Figure 4B and C**). Expression of the partially active mutant Lpa K159A produced a similar pattern of lipoylation to that observed in the other two active site mutants. This result is consistent with the similar growth rates observed in **Figure 4A** and indicates that Lpa K159A enzymatic activity is not significant enough to affect lipoylation *in vivo*.

Cells expressing the active site mutants had an approximately 10% reduction in lipoyl-PDH and 25% reduction in lipoyl-KDH compared to cells expressing empty vector (**Figure 4C**). This could be attributable to either residual activity in all of the mutants, or to lowered *kdh* and *pdh* expression in response to metabolic stress from the over-expression of Lpa constructs. In *E. coli*, *pdh* expression is repressed in response to the accumulation of metabolites such as acetyl-CoA and NADH [20]. We examined the activity of the Lpa active site mutants in a ^35^S-lipoic acid incorporation assay that allowed us to observe lipoylation of the H-protein as well as the PDH and KDH E2 subunits. The incorporation of radiolabel into the *E. coli* PDH and KDH was similar between the three active site mutants, while incorporation of radiolabel into the H-protein was difficult to assess due to its low signal (**Figure 4D**). Importantly, ^35^S-lipoic acid incorporation in cells expressing the active site mutants was similar to that in cells expressing the unrelated protein MBP-MCAT. Quantitative analysis of three independent experiments shows that lipoylation of the PDH, KDH, and H-protein does not significantly differ among cells expressing the three active site mutants or between cells expressing the mutants compared to cells expressing MBP-MCAT (data not shown). These results confirm that the three Lpa active site mutants do not have detectable lipoamidase activity, and that lower lipoylation levels observed in cells expressing these mutants are the result of metabolic stress associated with protein induction.

### Lpa toxicity in TM136 strain *E. coli*

**Figure 5:**
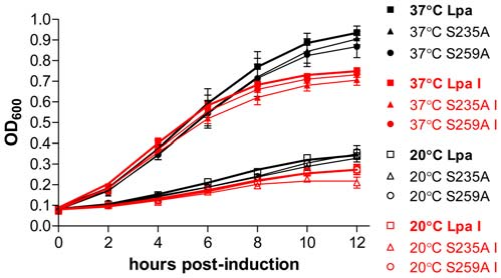
Growth of lipoylation deficient *E. coli* expressing Lpa and Lpa active site mutants. Growth assay of TM136 strain cells [16] expressing Lpa (squares), Lpa S235A (upward triangles), and Lpa S259A (circles). Growth curves at 37°C (solid symbols) and 20°C (open symbols are shown with cultures induced with 0.4 mM IPTG highlighted in red. In each condition, the growth curve corresponding to cells expressing wild type Lpa is shown with a thickened line.

The genes encoding the lipoate ligase (*lplA*) and the transferase (*lipB*) are disrupted in TM136 strain *E.coli*, preventing protein lipoylation [16]. Expression of Lpa in these cells will limit growth if Lpa interferes with any important metabolic pathways other than lipoylation. Expression of wild type Lpa in TM136 cells was compared to the active site mutants Lpa S235A and Lpa S259A. Triplicate cultures were initiated from an OD_600_ of 0.05 and maintained in four different conditions: 37°C with and without IPTG induction, and 20°C with and without IPTG induction. Cultures maintained at 20°C (**Figure 5**, open symbols) grew more slowly than their counterparts at 37°C (**Figure 5**, closed symbols), however, there was no significant difference between cells containing the Lpa plasmid *versus* those containing the active site mutants. Induction of protein expression with 0.4 mM IPTG slowed the growth of the cultures at both temperatures (**Figure 5**, red symbols), but there was still no significant difference in growth rate between cells expressing Lpa compared to those expressing the mutants. These data demonstrate that Lpa is only toxic to *E. coli* growth due to its lipoamidase activity, and that Lpa does not interfere with other important metabolic pathways in *E. coli*.

### Function and specificity of the amidase domain

**Figure 6:**
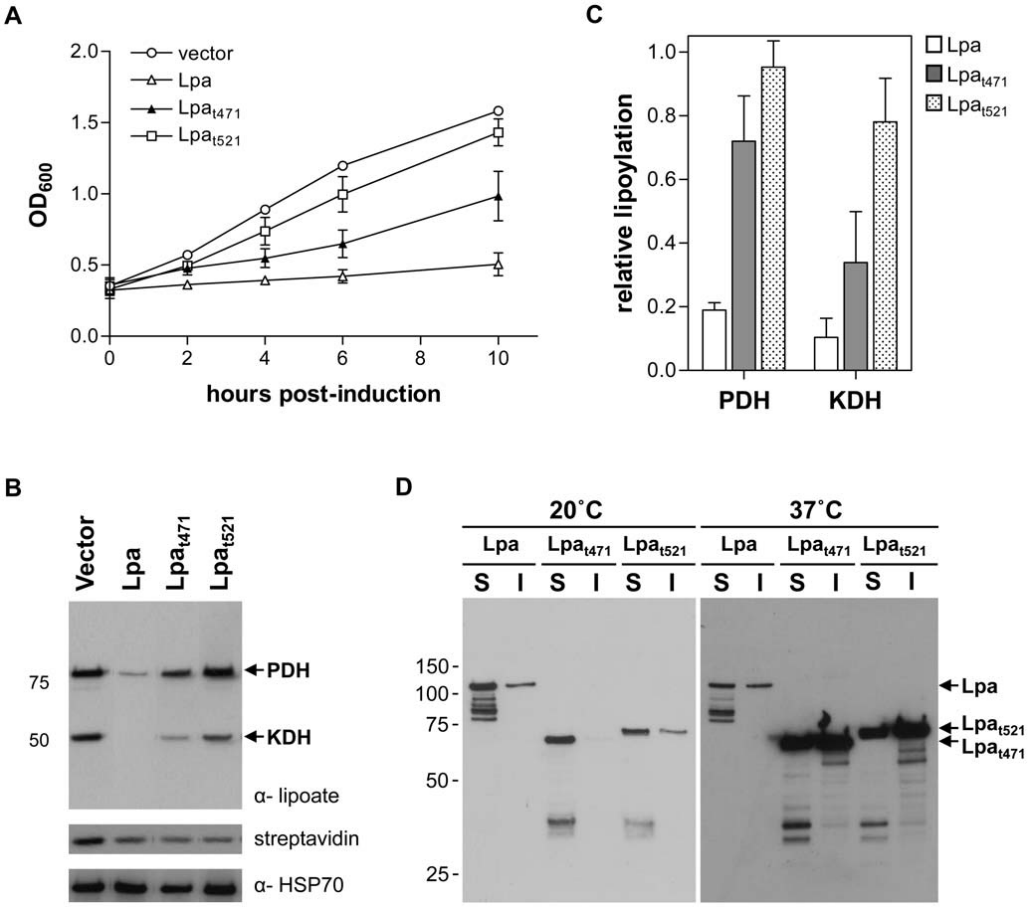
Growth and lipoylation of Lpa constructs containing only the amidase domain. (A) Growth assay of *E. coli* cells expressing Lpa_t471_ (closed triangles) and Lpa_t521_ (open squares) compared to expression of Lpa (open triangles) and vector alone (open circles). The OD_600_ of cultures was measured at the time of induction with IPTG and at 2, 4, 6, and 10 hours post-induction. Error bars represent the standard deviation of three replicates from a representative growth assay. (B) Anti-lipoic acid and streptavidin affinity blots to determine levels of lipoylation and biotinylation in *E. coli* expressing empty vector, Lpa, and Lpa truncation mutants. (C) Densitometry analysis of PDH and KDH lipoylation in cells expressing Lpa and Lpa truncation mutants. The fraction of lipoylation in cells expressing Lpa or the Lpa active site mutants relative to cells expressing vector alone was determined as described in Figure 4C. Error bars represent the SEM for the fraction of lipoylation for three independent western blots. (D) Solubility of Lpa and Lpa truncation mutants expressed at 20°C and 37°C. After induction of protein expression, cells were grown at 20°C for 10 hours or 37°C for four hours. Lpa in the insoluble (I) and soluble (S) fractions was analyzed by anti-His western blot.

Lpa is a two-domain protein, consisting of an amidase domain and a domain of unknown function. The second domain is of low complexity, and we explored whether the amidase domain alone had aminohydrolase activity. Two truncation mutants were made by introducing stop codons at codons 471 (Lpa_t471_) and 521 (Lpa_t521_) of the *lpa* gene. The Lpa_t471_ mutant contains the predicted amidase domain, whereas the Lpa_t521_ mutant contains an additional 50 residues of the second domain. When these constructs were expressed in *E. coli*, the Lpa_t521_ mutant did not inhibit bacterial growth, whereas the Lpa_t471_ mutant caused a moderate growth defect less pronounced than that of wild type Lpa (**Figure 6A**). Consistent with these effects on growth, quantitative western blot analysis shows that the PDH and KDH in cells expressing Lpa_t521_ are lipoylated at 95% and 78% of the empty vector level; these levels are similar to those observed in cells expressing the active site mutants (**Figure 4C**). In contrast, lipoylation in cells expressing Lpa_t471_ dropped to 72% (PDH) and 24% (KDH) of empty vector levels, indicating that Lpa_t471_ retains some aminohydrolase activity (**Figure 6B and C**). Cells expressing Lpa_t471_ contain about three times the amount of lipoylated PDH and KDH found in those expressing wild type Lpa. The differences in lipoamidase activity between Lpa, Lpa_t471_, and Lpa_t521_ are not attributable to differences in expression or solubility. At 20°C, there appears to be a significant pool of soluble Lpa_t521_ despite the lack of apparent activity for this construct (**Figure 6D**). The Lpa_t471_ construct is soluble and well expressed at both 20°C and 37°C, suggesting that this truncated form of Lpa could be an improved tool over wild type Lpa for probing α-ketoacid dehydrogenase inactivation *in vivo*. Biotinylation levels in cells expressing Lpa, Lpa_t471_, and Lpa_t521_ were similar, indicating that truncation of Lpa does not affect protein biotinylation (**Figure 6B**).

## Discussion

*E. faecalis* extracts containing lipoamidase have been used for several decades as a tool to probe the role of lipoic acid in α-ketoacid dehydrogenase enzymes [21]–[25]. Recently, Jiang and Cronan identified the enzyme responsible for lipoamidase activity and characterized the properties of this enzyme *in vitro* [9]. Cloning of the *lpa* gene made it possible to use lipoamidase as a tool to inactivate lipoylated enzymes in heterologous systems *in vivo*. In order to evaluate the effects of Lpa *in vivo*, we cloned the *lpa* gene into an *E. coli* expression vector which appends a hexa-histidine tag to the amino-terminus of Lpa. When expressed at 20°C, Lpa inhibits bacterial growth over the course of the 10 hour induction period (**Figure 1**). At 37°C, Lpa production peaked approximately four hours after induction, and normal bacterial growth resumed shortly thereafter. While the amount of Lpa at six and ten hours is diminished from the peak at four hours, a significant amount of Lpa protein persists but does not adversely affect bacterial growth. One possible explanation is that the *lpa* gene accumulates loss of function mutations similar to those previously observed [9], however, plasmids recovered from these experiments did not contain mutations. A more likely explanation is that the overall half-life of soluble, active Lpa is fairly short, and that the sustained presence of protein after peak induction at 37°C is due to the accumulation of insoluble and/or inactive protein. Indeed, **Figure 6D** shows that relatively more insoluble Lpa has accumulated after 4 hours at 37°C than after 10 hours at 20°C. Activity at physiologic temperatures, as demonstrated here, is crucial for the use of Lpa as an *in vivo* probe. In addition, the relatively short window of lipoamidase activity at 37°C is an attractive property since the activity of the probe could then be more tightly controlled in a conditional expression system.

The usefulness of Lpa as an *in vivo* probe of α-ketoacid dehydrogenase inactivation requires that the enzyme recognizes a broad range of lipoylated proteins. We show that Lpa cleaves radiolabeled lipoic acid from all three lipoylated proteins present in *E. coli* (**Figure 2**). Two of these proteins, the PDH E2 and the KDH E2, form very large multienzyme complexes that are several million Daltons in size [26]. The third protein, the H-protein, is approximately 14,000 Daltons, and does not form a stable complex with all other GCV components [27]. Thus, Lpa activity does not appear to depend on the overall architecture of the multienzyme complex, but rather on the recognition of the lipoylation domain shared by all lipoylated proteins.

The specificity of Lpa for lipoylated proteins versus other lysine modifications was examined in early work using partially purified *E. faecalis* extracts and small molecule moieties containing functional groups or cofactors attached to lysine through an amide bond [8]. These *in vitro* experiments showed that Lpa had a strong specificity for lipoylated proteins and small molecules. However, Lpa did hydrolyze ε-N-biotinyl-L-lysine (biotinyl-lysine), albeit at 2.5% the rate of ε-N-lipoyl-L-lysine hydrolysis [9]. *In vivo*, we did not detect hydrolysis of biotin from biotinylated proteins by affinity blot (**Figure 3**). This result demonstrates that Lpa does not have significant biotinidase activity *in vivo*, however, Lpa could catalyze this reaction at a rate much lower than the rate of biotin ligation. The opposite occurs for lipoylated proteins – Lpa cleaves lipoate more rapidly than the rate at which the cell is able to lipoylate proteins.

In a previous study, mutation of the Lpa active site residue K159 to alanine was found to result in partial activity, while the S235A and S259A mutants were found to be inactive [9]. We did not observe a difference in the growth of *E. coli* cells expressing these three Lpa active site mutations (**Figure 4**). Similarly, we did not detect significant differences in the lipoylation of PDH or KDH (as determined by western blot and autoradiography) or in the lipoylation of the H-protein (as determined by autoradiography). These results indicate that the three active site mutations have equivalent lipoamidase activity in our *in vivo* assays. Although unlikely, it is possible that the three mutants have an equivalent low level of lipoamidase activity. Indeed, this appears to be the case based on the observation that cells harboring the empty expression vector grow more rapidly and contain higher levels of lipoylated proteins than cells harboring any of the three active site mutants (**Figure 4**). This effect, however, is triggered by the metabolic stress associated with protein expression, which is largely absent from cells expressing only the empty vector. When an unrelated protein (MCAT) was expressed, lipoylation was indistinguishable from that observed with the three Lpa active site mutants. Taken together, these results show that three Lpa active site mutants do not have detectable activity in our *in vivo* assays.

Lipoamidase interferes with the lipoylation of all three lipoate dependent proteins in *E. coli*, and it could also interfere with other important metabolic pathways. To address this question, we assessed the growth inhibition associated with expressing Lpa in an *E. coli* strain which lacks lipoate metabolism. In strain TM136 *E. coli*, the genes encoding lipoate ligase (*lplA*) and the lipoyl(ocantoyl)-acyl carrier protein:protein transferase (*lipB*) are disrupted through transposon mutagenesis (*lipB*::Tn1000dKan *lplA*::Tn10) [16]. These cells, which were a kind gift from John Cronan, do not contain lipoylated proteins, but will grow in culture medium supplemented with acetate and succinate to bypass the loss of lipoylated PDH and KDH, respectively. We analyzed the growth of TM136 cells transformed with plasmids encoding either Lpa or Lpa active site mutants (**Figure 5**). The presence of Lpa did not decrease the growth rate of these cells as compared to the active site mutants. This was true at 37°C and at 20°C, regardless of whether protein expression was induced with IPTG. These results prove that Lpa toxicity in *E. coli* is solely manifest through interference with lipoate metabolism.

The carboxy-terminal domain of lipoamidase is not required for activity. We generated a mutant Lpa truncated after the predicted amidase domain at residue 471 (Lpa_t471_). This mutant caused a partial growth defect and retained Lpa activity as tested by anti-lipoate western blot, however, a variant extending to residue 521 (Lpa_t521_) did not interfere with growth or lipoylation (**Figure 6**). This construct is expressed at levels similar to those observed with Lpa_t471_ and it is only slightly less soluble. Perhaps the additional 50 amino acids in the Lpa_t521_ construct interfere with substrate binding, limiting its activity *in vivo*. Growth assays and western blot analysis show that Lpa_t471_ is less active than wild type Lpa; however, its activity is sufficient to cause a growth defect and measurable decrease in lipoylation, making Lpa_t471_ a potentially useful probe in other organisms. Importantly, truncation of the carboxy-terminal domain does not affect protein biotinylation, suggesting that the second domain is not essential for activity or specificity. The smaller size of Lpa_t471_ may make it more amenable to expression in heterologous systems.

**Figure 7:**
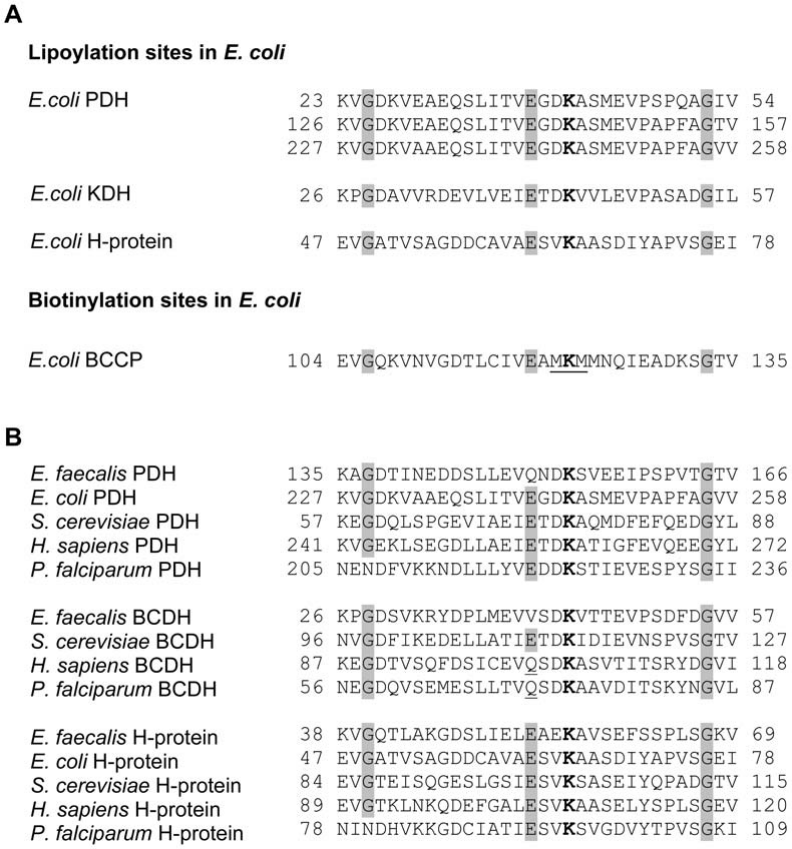
Lipoylation and biotinylation sites. (A) Amino acid sequence alignments of lipoylation and biotinylation sites in *E. coli*. The lysine that is involved in lipoic acid or biotin attachment is marked in bold. Residues corresponding to conserved glycine and glutamine residues are shaded [33]. Residues forming the biotinylation consensus site are underlined. (B) ClustalW comparison of amino acid sequences surrounding the site of lipoate attachment for lipoylated proteins found in *E. faecalis*. The substitution of the Glu three residues amino-terminal to the lipoyl lysine with Gln (underlined residues) is a common motif in the BCDH E2.

Two factors may allow Lpa to discriminate between lipoylated proteins and biotinylated proteins despite the fact that the cofactors are similar in structure and the proteins that they are attached to share the same protein fold. NMR structures of the *E. coli* BCCP show that the biotin cofactor is sequestered by a thumb-like region [28], making the biotin-amide bond less accessible to Lpa than on biotinyl-lysine. Also, the residues immediately surrounding the biotinylation site are well conserved among biotinylated proteins, and distinct from those found in analogous positions in lipoylated proteins (**Figure 7A**). These residues help biotin ligases and lipoate ligases to discriminate between possible substrates and may be important for lipoamidase specificity as well. Substitution of the MKM motif found in the *E. coli* BCCP with a sequence commonly found in lipoylated proteins (DKA) resulted in partial lipoylation of the BCCP [29]. Mutation of the residue preceding the biotinylation site in yeast pyruvate carboxylase 1 compromises biotinylation [30]. Similarly, mutation of the glutamate three residues before the lipoylation site to glutamine in human PDH prevents lipoylation [31].

The role that lipoamidase plays in *E. faecalis* is not clear. Genes encoding PDH, BCDH and H-protein can all be found in the *E. faecalis* genome, however, lipoate is not required for anaerobic growth when a sufficient source of fermentable material is available [32]. Under conditions favoring aerobic growth, lipoylation of PDH and BCDH may be important. It is interesting to note that the residues found in the *E. faecalis* PDH and BCDH lipoylation domains are distinct from those found in other organisms, while the residues found in the *E. faecalis* H-protein are identical to other organisms at the conserved amino acid sites (**Figure 7B**). As in *E. coli*, in *E. faecalis* the H-protein may not be necessary for growth under aerobic conditions, and the use of divergent lipoylation site sequences for the PDH and BCDH may help to limit the activity of lipoamidase against these proteins in *E. faecalis*.

Lipoamidase is a potentially useful tool for probing the role of lipoylation *in vivo*. Lpa inactivates a broad range of lipoylated proteins and does not seem to interfere with any other important metabolic pathways in *E. coli*. The mutant K159A, which is partially active *in vitro*, did not show detectable activity *in vivo*, indicating that the three active site mutants, K159A, S235A, and S259A, can be used as inactive controls in an *in vivo* experiment. Lpa activity can be tightly controlled by a conditional expression system and may be especially useful in probing lipoate metabolism in organisms in which gene disruptions and gene knockdowns are impractical or impossible. A major limitation of Lpa as an *in vivo* probe is whether the organism of interest is able to express Lpa because of its large size and the low complexity of the second domain. The engineering of Lpa_t471_ reduces the length of Lpa by 259 amino acids and increases the chance that the probe can be expressed in active form in heterologous systems. Taken together, these studies in *E. coli* demonstrate how lipoamidase can be employed to probe lipoate metabolism *in vivo*.
